# A Multimodal IMU-RTLS Manufacturing Motion Dataset for Worker Activity Recognition

**DOI:** 10.3390/s26206665

**Published:** 2026-10-21

**Authors:** Andrea Sbaragli, Matteo Nardello

**Affiliations:** 1 School of Engineering, Newcastle University, Newcastle Upon Tyne NE1 7RU, UK; 2 Department of Industrial Engineering, University of Trento, Via Sommarive 9, 38123 Trento, Italy; matteo.nardello@unitn.it

**Keywords:** worker activity recognition, RTLS, IMU, machine learning, human-centric manufacturing

## Abstract

Worker Activity Recognition can improve operational visibility in human-centric manufacturing systems. However, existing approaches remain limited by scarce industrial datasets, isolated task recordings, and sensor-intensive configurations. This study presents two multimodal datasets and a cyber-physical architecture for complementary levels of worker motion. The Gross Worker Activity Recognition dataset targets operator proximity to workstation and the identification of logistic activities in reconfigurable manufacturing systems. It contains 814,065 positioning samples from eight production setups, each repeated twice, involving more than 40 workers. This dataset is benchmarked with different learning structure algorithms, and the Long Short-Term Memory is selected as a baseline model, achieving an F1-score of 86%. The Fine Worker Activity Recognition dataset targets the segmentation of five pump-assembly operations using two inertial measurement units and a radio-frequency-identification smart glove. It comprises approximately 7 h of recordings and more than one million samples from 50 complete assemblies performed by three workers. Transformer, ResNet, and SqueezeNet models are benchmarked, with SqueezeNet selected as the baseline after achieving the highest F1-score of 85.2%. Moreover, the results identify transition-rich activities as the principal recognition bottleneck and show how complementary motion levels support different manufacturing indicators.

## 1. Introduction

Industry 5.0 positions workers at the centre of next-generation manufacturing systems [1]. This approach is increasingly relevant as population aging reduces workforce availability and increases the need for adaptable production environments [2]. In this context, Internet of Things (IoT) wearable machine learning (ML) algorithms enable operations and workforce resilience monitoring [3,4]. The resulting data streams improve process visibility, support task and resource allocation, enable the early identification of operational deviations, and facilitate coordinated human–machine responses to changing production conditions [5].

Among the different manufacturing applications, Worker Activity Recognition (WAR) is gaining a lot of traction due to its ability to identify and segments workers’ tasks executions [6]. The resulting activity data enable the extraction of key performance indicators (KPIs) related to workstation assignment, logistics events performance, resource utilisation, and process efficiency across different layouts and production batches [7]. Manufacturing WAR applications comprises two complementary levels that differ in motion granularity and sensing technology [8]. On the one hand, gross WAR uses Real-Time Locating Systems (RTLSs) to track inter-workstation movements and material transport [9,10]. On the other hand, fine-grained WAR uses Inertial Measurement Units (IMUs) or motion-capture cameras to recognise task-level actions, such as component picking, tool use, screwing, and manual assembly [11,12]. Despite the development of different cyber-physical systems (CPS) for this domain, research remains limited at both recognition levels. Gross WAR rarely incorporates ML models, limiting cyber-layer transferability across manufacturing applications and requiring use-case-specific configurations. This dependence also reduces adaptability to variations in worker motion and sensor noise [13]. In contrast, fine-grained WAR often relies on costly multi-sensor configurations and short, isolated recordings that omit complete activity sequences and natural task transitions [14]. Overall, these limitations constrain the transfer, scaling, and deployment of WAR solutions in operating manufacturing environments.

To address these limitations, this paper presents two multimodal, manufacturing-oriented datasets as its core contribution:

- **RMS Gross WAR:** This dataset validates the operator assignment to workstations and identifies logistic activities in human-centric RMS. The RMS layout and operators’ indoor positions are acquired by an Ultrawide Band RTLS and fed into a ML-based computational unit to segment workers’ activities. The Long Short-Term Memory (LSTM)-based neural network is benchmarked with a clustering and tree-based algorithm and is the most performing algorithm, providing an F1-score equal to 86%. This gross dataset has been validated with eight production set-ups performed twice, involving more than 40 workers.
- **Assembly Fine WAR:** This second dataset targets segment fine-grained screwing and manual activities during the assembly of a centrifugal pump in one industrial workstation. Two IMUs capture workers’ motion patterns during the assembly process, whereas an RFID-enabled smart glove detects interactions when items and components are picked from the supermarket. This dataset benchmarks a Transformer with two CNNs, where the SqueezeNet emerges as the most performing model with an F1-score of 85.2% in classifying five common assembly operations. This assembly-driven digital solution is validated in a real-world deployment involving three workers assembling a multistage centrifugal pump across 50 repetitions, achieving more than 7 h of recording and one million datapoints.

Besides the public release, the datasets establish a reproducible benchmark for investigating two unresolved aspects of industrial WAR: generalisation across reconfigurable layouts and the recognition of heterogeneous transition-rich activities. The accompanying CPS provides a modular mapping from sensing granularity to ML classification and decision support. This structure integrates gross motion, fine-grained activities, and process interactions into time-driven events that support production and worker-resilience monitoring. Together, the datasets and CPS provide the empirical and technical foundation required to extend, validate, and progressively scale WAR applications across manufacturing environments.

The remainder of this paper is structured as follows. Section 2 reviews gross and fine WAR applications in human-centric manufacturing systems. Section 3 presents the two multimodal datasets. A modular CPS is presented in Section 4 covering IoT data acquisition, algorithms, and a decision-support system to scale fine and gross WAR in manufacturing applications. Section 5 presents different ML algorithms for the presented datasets. Finally, Section 6 outlines the conclusions and further research directions.

## 2. Related Work

This Section reviews WAR application focusing on sensing architectures, recognition performance, and limitations concerning robustness, generalisation, cost, and industrial deployment. While Section 2.1 targets gross motion monitoring during task execution, the fine level is discussed in Section 2.2.

### 2.1. Gross Worker Activity Recognition Applications

RTLSs are widely adopted to continuously track workers task and motion patterns in human-centric manufacturing environments [15]. Several positioning techniques and communication protocols have been proposed and validated to determine the unknown position of tags with respect to fixed anchors [16]. Positioning approaches generally fall into fingerprinting-based or geometric-based methods, with Time Difference of Arrival (TDoA) emerging as a widely adopted solution in industrial environments. TDoA estimates a tag’s position by computing the intersection of hyperbolic curves derived from signal arrival time differences at three or more anchors [17]. In terms of communication technologies, ultrawide-band (UWB) stands out for its high localisation accuracy, outperforming optical, infrared, and conventional radio frequency systems due to its large bandwidth—at least 500 MHz—which enables fine multipath resolution [16,17].

Within manufacturing environments, RTLSs support different WAR applications. For worker safety, positioning datastreams enable real-time location monitoring to protect personnel, reduce emergency response times, and simplify time reconciliation through wearable tags on visitor cards, clothing, or helmets [18]. Similarly, ref. [19] proposes a framework to optimise automated-guided-vehicle routes based on future workers’ positions and therefore avoid in-plant collisions. Beyond safety, human-centric RTLS application remains very limited. Many CPSs monitor other industrial assets such as work-in-progress products to identify production bottlenecks and throughput times [10,20,21]. However, these applications predominantly rely on geofencing algorithms, exposing CPS to inherent measurement noise and limiting result replicability across production shifts. Recent advances in ML-based computations are addressing this replicability gap in RTLS manufacturing applications. For instance, ref. [22] feeds an RTLS Received Signal Strength Indicator into an ML layer, achieving indoor positioning accuracies that exceed 70%. However, this CPS predominantly focuses on asset positioning rather than WAR and evaluations on the system’s and operator’s resilience.

Overall, current RTLS-based WAR research remains constrained across three dimensions. These limitations can be summarised as follows:

- **Application:** Human-centric investigations remain largely confined to worker safety, with limited consideration of performance oriented WAR applications.
- **Computation:** The predominant reliance on geofencing makes activity inference sensitive to measurement noise and dependent on use-case-specific spatial configurations.
- **Reproducibility:** The lack of publicly available datasets restricts independent validation, transfer learning opportunities, and benchmarking across manufacturing contexts.

### 2.2. Fine Worker Activity Recognition Applications

Fine WAR applications are widely ML intense and usually digitise workers’ task execution using motion capture cameras (MOCAP) and IMUs [11]. For example, ref. [23] leverages a single Kinect node to track 20 workers’ body joints during task execution. These time-dependent spatial data are fed into different configurations of a hidden Markov model to classify assembly tasks, ranging from painting to loading. The model also addresses operational uncertainties inherent in manufacturing cycles—such as worker-to-worker coordination delays and unscheduled machine maintenance—by incorporating an “unknown” class to capture activities that fall outside predefined categories. This intrinsic variability of the human factor deteriorates the computational algorithm’s performance to 67%, affecting the successful deployment of this CPS in real operating environments. Besides the performances, this approach suffers from two other major limitations. First, the MOCAP measuring unit is susceptible to occlusions, requiring multiple synchronised nodes. This aspect massively increases the cost of ownership, potentially leading to low adoption across manufacturing plants. Second, manual data removal is required due to the light reflection of industrial equipment. This provides repeated false skeleton detections, placing the worker under the conveyor or even the floor. The need to address the human factor entropy is further reiterated by [24]. This human-centric CPS leverages wrist-worn IMUs to classify manual operations and task transitioning in a meat processing plan. Acceleration and gyroscope data streams are fed into an Support Vector Machine (SVM) to classify various activities, from idle states to separating meat. However, this production-ready CPS fails to generalise fine motion patterns. Indeed, the ML-based classifier performances for a single worker and four combined peaked at 70% and 63%, respectively. As discussed above, these metrics prevent the adoption of fine WAR CPS, as they would lead to skewed KPIs and thus an under-performing decision-making process. Classification improvements can be found in the literature only when capturing data streams in isolated tasks. For instance, ref. [14] proposes a multi-modal CPS that fuses IMU-based armband and cameras to digitise workers’ activity execution and the industrial environment. These motion time-series are fed into a Convolutional Neural Network (CNN) with multiple layers to classify six basic assembly activities, such as tool usage and resting scenarios, achieving over 90% accuracy. However, the validation campaign strictly confines this application within academia, as tasks are recorded individually rather than within full assembly sequences. The human-centric manufacturing environment presents high variability during task execution, and therefore, it is imperative to model workers’ variability in transitioning to the next task. The SKODA dataset partially addresses these limitations by monitoring human-centric car inspection tasks using 20 IMUs placed on upper limbs [25]. Monitored tasks include opening and closing of several car parts, such as the trunk and doors, including also the “null” class that models the intrinsic variability of the human factor in transitioning from one task to the next one. In this regard, ref. [26] designs a deep convolutional LSTM-based neural network, achieving an F1-score around 95%. Despite these production-ready performances, generalisation is very limited as the experimental campaign involved solely one subject performing 70 repetitions for each task. In addition, the high number of sensing nodes may affect operators’ comfort during task executions, limiting the adoption in production-ready scenarios. Cost represents an additional downside of the proposed CPS that linearly increases with larger shop floors.

Overall, manufacturing-oriented fine WAR remains constrained across the following three dimensions:

- **Reproducibility:** Although public datasets exist for broader human activity recognition [27,28], industrial multimodal datasets remain scarce, limiting benchmarking and cross-study validation.
- **Production readiness:** The vast majority of applications contain isolated task recordings that omit complete assembly sequences and natural transitions between activities.
- **Sensing:** Improved classification performance often requires complex multi-sensor configurations, increasing deployment costs, system complexity, and operator burden.

To fill limitations of both gross and fine WAR applications, the following Sections discuss the WAR datasets by providing multimodal measurements and the CPS design for realistic industrial environments deployments.

## 3. Worker Activity Recognition Datasets

The proposed WAR datasets are collected through two experimental campaigns designed to capture workers’ motion patterns in real manufacturing environments. The datasets cover two complementary levels of motion, requiring different sensing technologies and sampling frequencies, as summarised in Table 1. On one hand, the RMS Gross WAR dataset targets the detection of workers’ proximity to production resources and logistic activities using an RTLS network composed of six anchors, covering an area of up to $250\;m^{2}$. On the other hand, the segmentation of manual assembly operations using IMUs and an RFID-based smart glove is addressed by the Assembly Fine WAR dataset. This level of analysis provides higher temporal and motion resolutions with reduced deployment cost compared to UWB-based RTLS process monitoring (see Table 1). Further details on the acquisition systems and sensing configurations are discussed in Section 4.

### 3.1. RMS Gross Worker Activity Recognition

**Figure 1:**
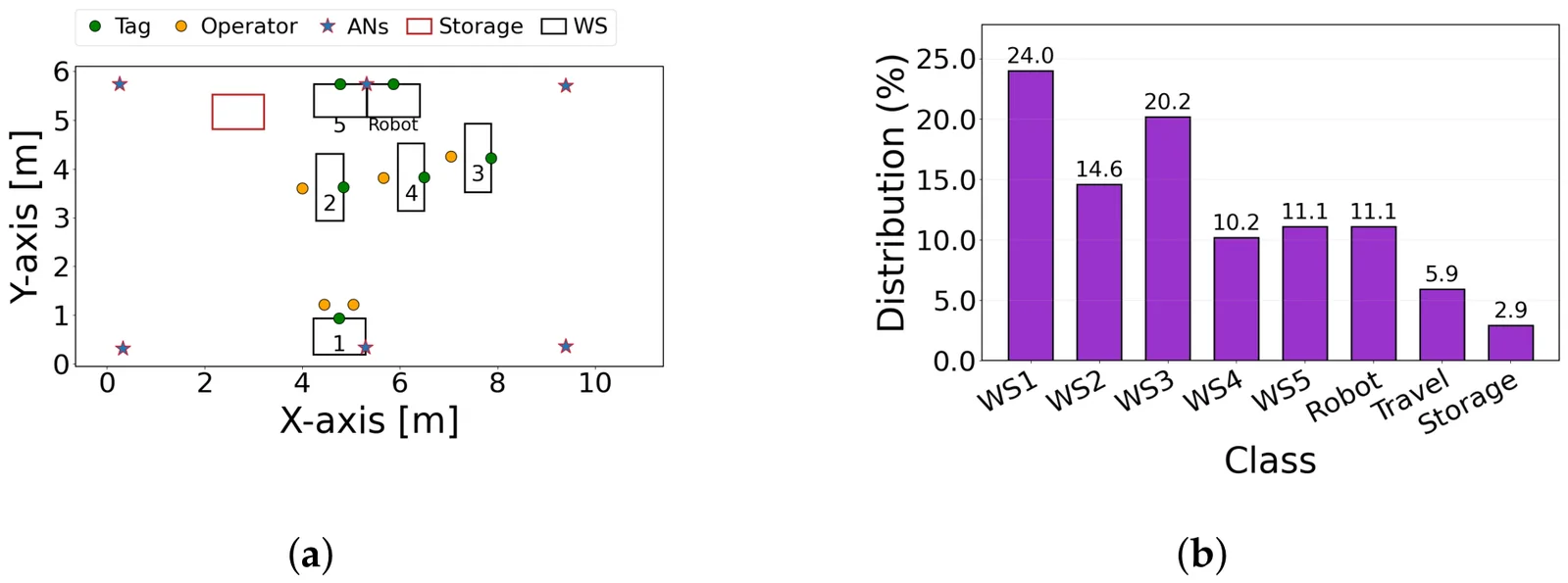
RMS Gross WAR dataset [13]: (**a**) industrial environment; (**b**) class distribution.

The Gross WAR dataset focuses on gross motion recognition in a human-centric RMS. The RMS adjusts its design and functioning cost effectively based on the manufacturing output, involving, for instance, different layout configurations and specific task assignments to resources [29]. The experimental campaign monitors the assembly of a medical spinal prosthetic using multiple industrial resources, including a collaborative robot. Figure 1a shows the layout of production setup 7, which involves five active workers, together with the positions of the RTLS anchors (ANs). Resources includes workstations (e.g., WS) equipped with industrial screwdrivers and a storage location containing components and fasteners. The assembly process consists of several parallel and sequential tasks with a total duration ranging from 15 to 20 min, depending on teams’ composition and worker expertise. The prosthetic’s blocks are paired and glued, drilled, before being fitted and fastened into products frames with industrial drivers. The final stage involves joining prosthetic halves and performing quality checks. Eight different production setups are evaluated, varying workstation configurations, task allocation, and workforce composition (see Table A1). Each setup is performed twice on different days, involving 40 workers and resulting in 814,065 human-centric positioning samples. The resulting imbalanced dataset is presented in Figure 1b, where classes represent proximity to specific workstations (e.g., WS1, WS2, etc.), robotic WS (e.g., Robot), storage area (e.g., Storage), and logistic movements (e.g., Travel class). Ground-truth labels are established from camera recordings of each production setup. A single annotator reviews all recordings and marks, for each operator, intervals of proximity to industrial resources (e.g., WS1, Robot, and Storage) and movement between resources (Travel). The timestamp marking the start of each camera recording provides the reference for aligning these intervals with the RTLS measurements. Where the boundary between resource proximity and Travel is unclear, the annotator examines the footage frame by frame to identify the visible transition. A Python (e.g., v3.11) script maps the annotated intervals to the timestamped RTLS samples and assigns the corresponding class labels.

### 3.2. Assembly Fine Worker Activity Recognition

**Figure 2:**
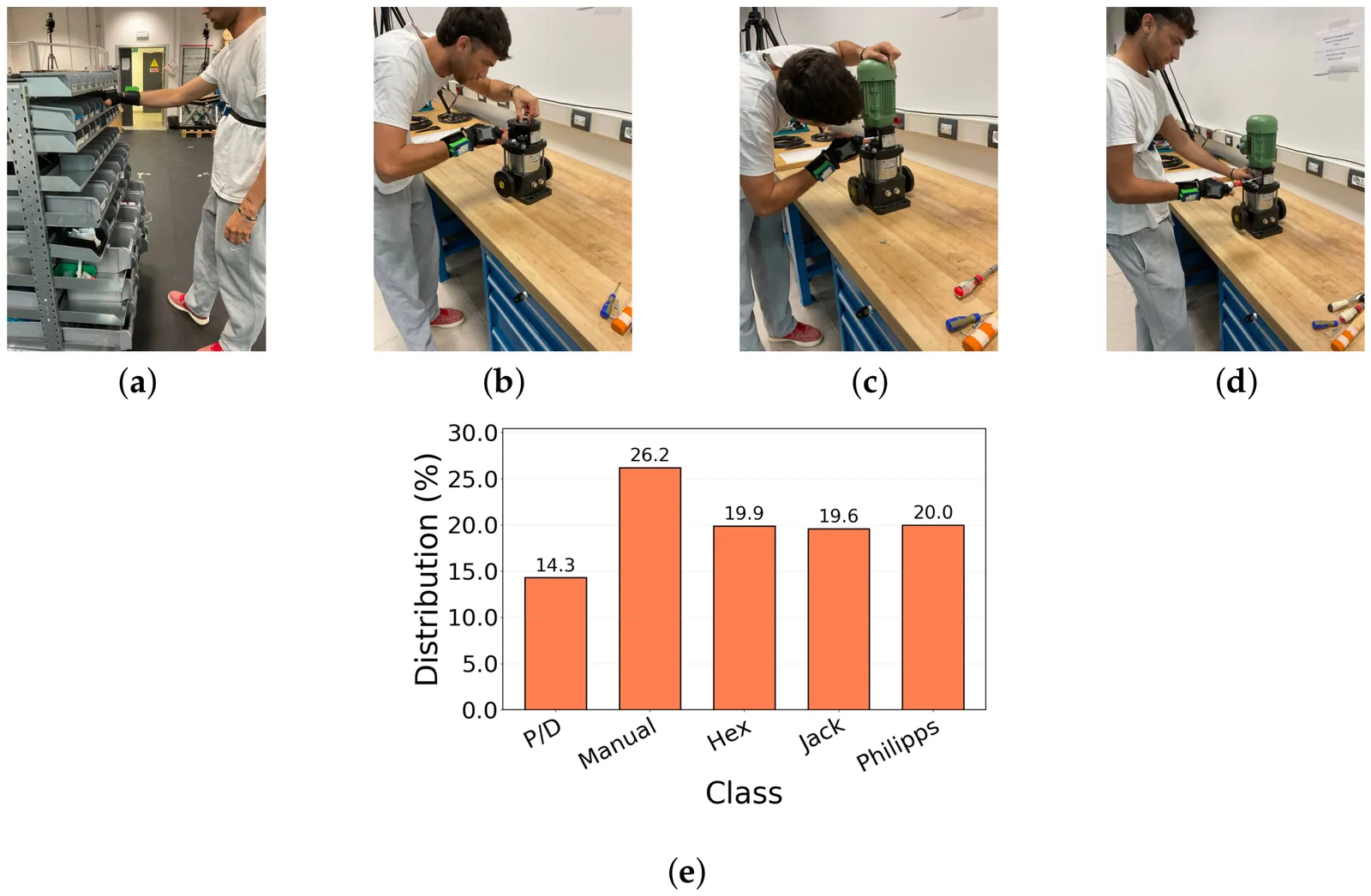
Assembly Fine WAR dataset [30]: (**a**) picking/depositing operation; (**b**) hexagonal screwing operation; (**c**) jack screwing operation; (**d**) Phillips screwing operation; and (**e**) class distribution.

The Assembly Fine WAR dataset targets the detection of fine-grained assembly activities, involving the assembly of a multistage centrifugal pump in a pilot industrial environment. Each assembly cycle lasts between 5 and 7 min. The assembly sequence is divided into three main activities (see Figure 2) [30]:

- **A1—Bush Mounting:** An operator starts the assembly cycle by picking the pump base from the ground and fasteners and bush from the supermarket in Figure 2a. An initial manual fitting, followed by a hexagonal screwing operation, partially tightens the bush within the pump housing (see Figure 2b). This task takes an average of 1.45 min (±0.41 min).
- **A2—Motor Mounting:** Following the retrieval of the components and the ground motor from the supermarket, the unit is fitted into the pump housing, and the bush is permanently secured using the same hexagonal screwdriver. Four bolts are then manually inserted into the motor base and tightened using the jack screwdriver, as shown in Figure 2c. This is the most time-consuming activity in the sequence, with an average duration of 2.52 min (±0.91 min).
- **A3—Plates Mounting:** Two aluminium plates are aligned and secured on each side of the pump using a Phillips screwdriver, after which the finished product is deposited back in the supermarket area (see Figure 2d). This task has an average duration of 1.23 min (±0.37 min).

Three workers perform multiple repetitions of the full assembly sequence, resulting in approximately 7 h of recordings and more than one million samples. The participants have the following anthropometric characteristics: Participant 1 is 178 cm tall and weighs 76 kg, Participant 2 is 185 cm tall and weighs 80 kg, and Participant 3 is 192 cm tall and weighs 90 kg. The full-assembly dataset comprises five recording days. On each day, Participants 1 and 2 each complete five assembly cycles, yielding 25 cycles per participant. Participant 1 is recorded before Participant 2, with no overlap between their recording intervals. To mitigate class imbalance, Participant 3 completes a separate 45 min session alternating among picking and depositing components and products and the Hexagonal, Jack, and Phillips operations. Figure 2e depicts the class distribution, where P/D and Hex groups picking and depositing activities and hexagonal operations, respectively. The Manual class aggregates a diverse set of intermediate actions, including bolt alignment, screw handling, and transitions between tool usages. The high variability and entropy of the associated motion patterns may pose a challenge to the learning capabilities of ML algorithms. However, capturing these transitional behaviours remains essential for deploying WAR-oriented CPS in real-world industrial environments. Ground-truth labels are established from camera recordings of the pump assemblies and the focused basic-operations session. A single annotator reviews all recordings and marks intervals of P/D, Hex, Jack, and Phillips operations, as well as intermediate actions and transitions assigned to the Manual class. The start timestamp of each camera recording provides the temporal reference for aligning these intervals with the sensor measurements. Where the boundary between activities is unclear, the annotator examines the footage frame by frame to identify the visible transition. A Python script maps the annotated intervals to the timestamped sensor samples and assigns one of the five activity labels to each sample.

Based on this dataset discussion, the following Section discusses the design of the acquisition layer and baseline ML algorithms for both levels of motion.

## 4. Cyber-Physical System for Worker Activity Recognition

**Figure 3:**
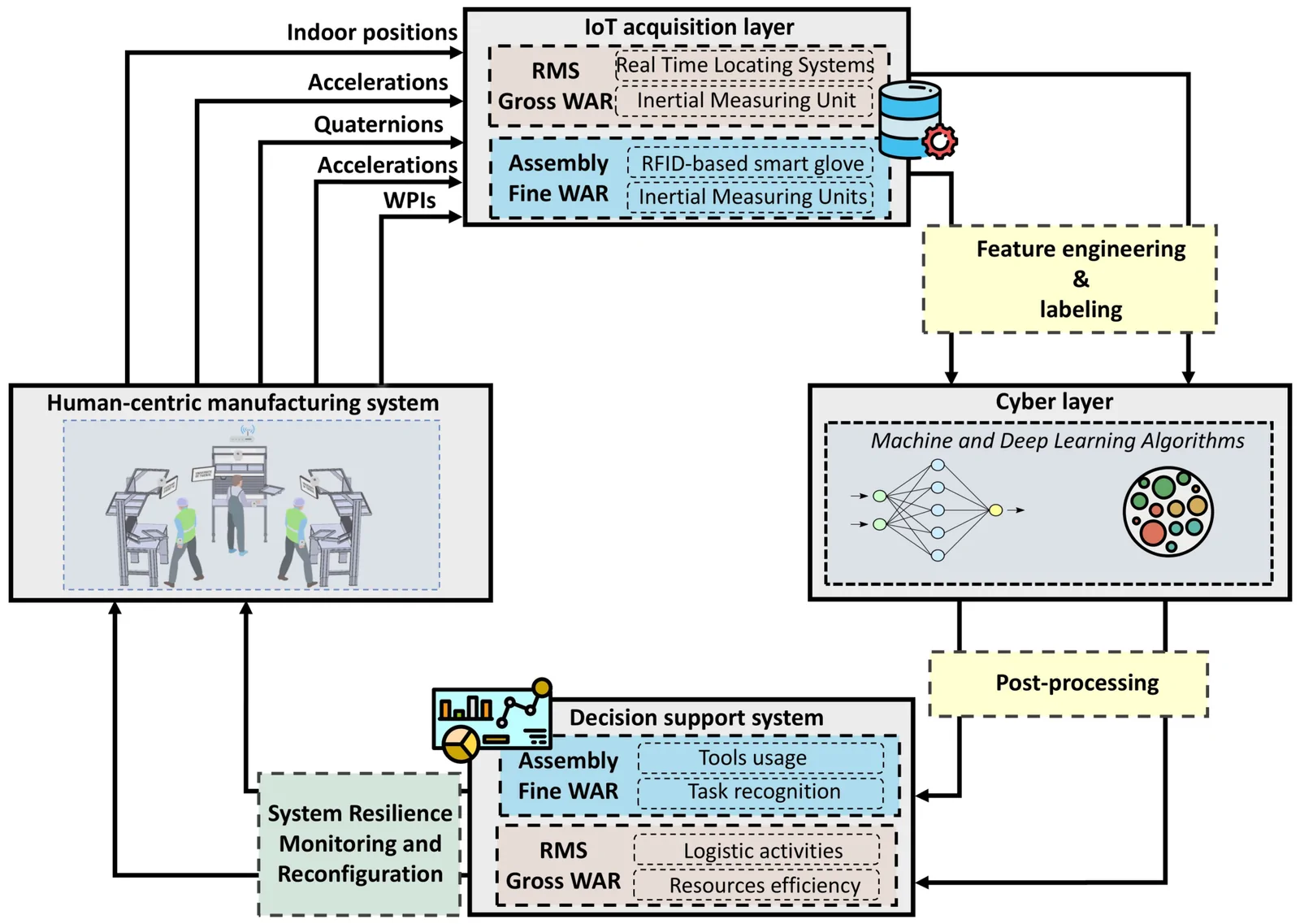
Cyber-Physical System overview for Worker Activity Recognition [30].

This section presents the CPS implementation to deploy comprehensive WAR applications in human-centric manufacturing systems (see Figure 3) [30]. Besides the monitored industrial system, the architecture consists of three entities. The IoT acquisition layer digitises workers’ task execution for both level of motion. On the one hand, the gross level is monitored via an RTLS network of six anchors, and both industrial resources and operators are equipped with tags to monitor their positional information across production shifts. On the other hand, two IMUs and an RFID-based smart glove monitor fine motion patterns in assembly tasks. These multi-dimensional data streams are fed into the cyber layer, benchmarking different ML algorithms. These baseline models process different input data representations and adopt different learning mechanisms, such as convolutional and recurrent operations. Following post-processing, the classified fine- and gross-motion patterns are integrated into a decision-support system as operational KPIs, providing visibility into system and worker resilience.

### 4.1. IoT Acquisition Layer

This section presents a modular sensing architecture that independently monitors both WAR levels and integrates their outputs to provide higher manufacturing visibility with limited intrusiveness. The selected hardware balances sensing performance and deployment cost without relying on sensor-dense acquisition configurations.

#### 4.1.1. Design for Gross Worker Activity Recognition

**Figure 4:**
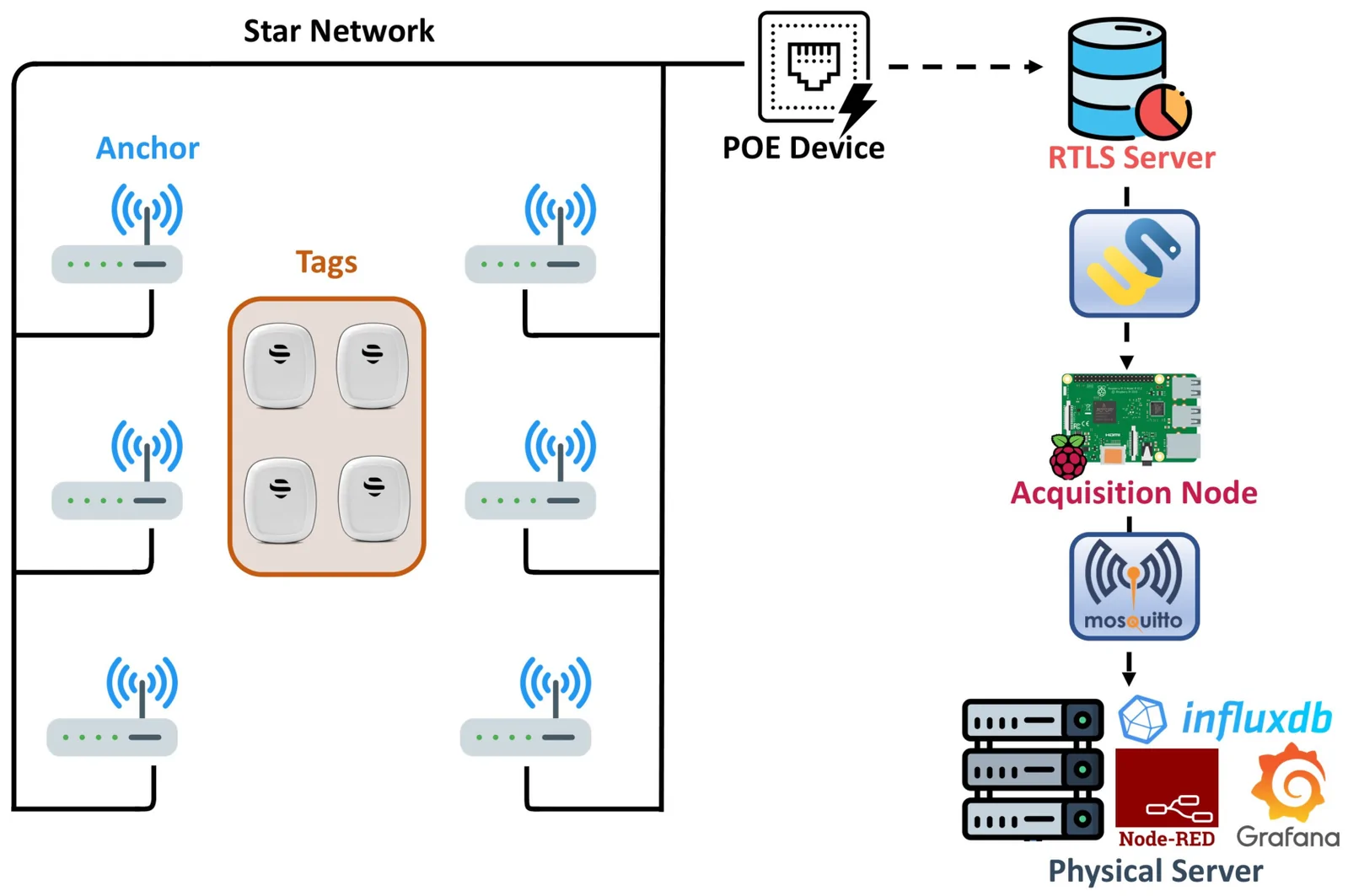
Design of the RTLS network for RMS Gross Worker Activity Recognition.

The gross motion acquisition layer relies on a UWB-based RTLS network to collect workers’ spatial information and workstation positions across production setups. These data are used to derive WS proximity and logistic activities. Six anchors define the reference system and are installed on the ceiling at known locations, covering an area of approximately $70\;m^{2}$. The anchors are based on Qorvo modules with IEEE-compliant UWB transceivers and are powered through a Power over Ethernet (PoE) switch, forming a star network topology (see Figure 4). Worker and workstation positions are estimated using the TDoA method, and measurements are stored as JSON messages on the RTLS server. Two types of positioning tags are deployed. Workers wear anonymous tags on the wrist of the dominant hand, sampled at 10 Hz. Workstations are tracked at 1 Hz using tags placed at fixed locations. This difference in sampling frequency reflects the higher dynamics of worker motion compared to static industrial resources. Each measurement contains a date and time timestamp, a tag ID identifying the operator or workstation, and three-dimensional (*x*, *y*, *z*) coordinates, although the *z* coordinate is excluded from the analysis. To enable external data processing, positioning data are streamed from the RTLS server to an external node. A Raspberry Pi 4 subscribes to the RTLS data via WebSockets and republishes them using the MQTT protocol over a TLS-encrypted Mosquitto broker. A server node processes the incoming data using Node-RED and stores time series in an InfluxDB database (see Figure 4).

#### 4.1.2. Design for Fine Worker Activity Recognition

**Figure 5:**
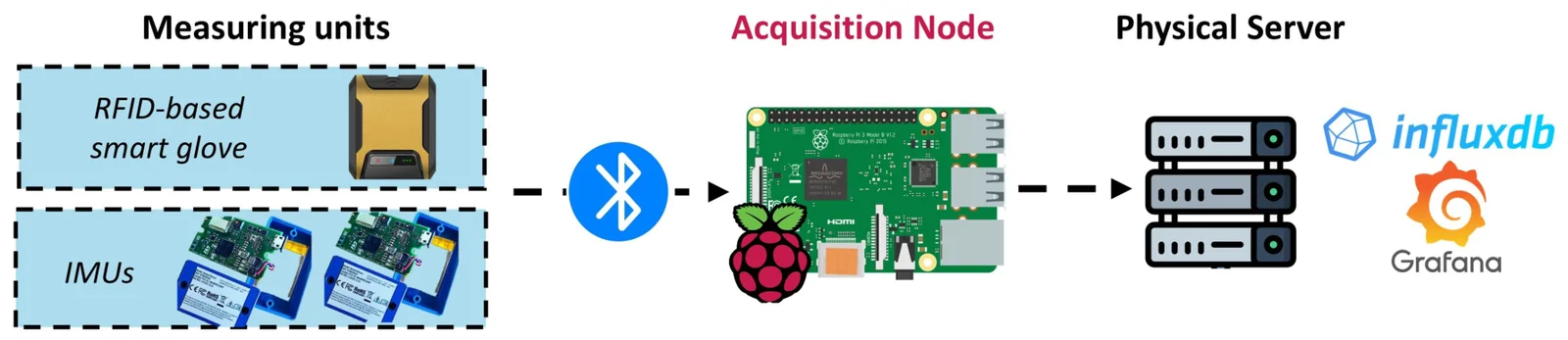
Design of the acquisition layer for Assembly Fine Worker Activity Recognition [30].

Fine motion is monitored using three wearable sensors, as shown in Figure 5 [30]. Data acquisition is managed by a Raspberry Pi 4, which handles concurrent Bluetooth connections with the sensors and ensures continuous data collection. Inertial data are transmitted in one-minute recording batches by the client node to an InfluxDB instance hosted on a local server within the same network. This modular and low-cost architecture allows multiple sensing devices to be integrated with a single acquisition node. Two IMUs capture tri-axial acceleration and orientation data. One sensor is placed on the dominant hand to capture detailed motion, while the second is positioned on the upper back to track body posture. Each sensor integrates a low-power STM32L4 microcontroller with an accelerometer, gyroscope, and magnetometer, streaming data at 52 Hz. An RFID-based smart glove records process interactions during picking and depositing operations. The glove includes a battery-powered tag reader containing a Cortex-M3 microcontroller paired with a linearly polarised antenna. Power output can be modulated within a 1 to 30 dBm range, yielding a maximum reading distance of 80 cm. This sensing node operates at a sampling frequency of 10 Hz, collecting the unique tag ID, the received signal strength indicator (RSSI), and the timestamp for each scanned passive RFID tag within range. Weighing 64 g in total, the unit is configured for continuous industrial application without restricting operator movement. The acquisition system supports both offline processing and near-real-time visualisation through a Grafana dashboard. This dual-placement approach ensures modular and non-intrusive monitoring of fine and gross activities using a limited number of measuring units.

### 4.2. Cyber Layer

This computational unit converts RTLS and IMU measurements into ML-ready training data representations. The following subsections describe the input representations, preprocessing procedures, and the benchmarked classification algorithms for each WAR level.

#### 4.2.1. Machine Learning for Gross Worker Activity Recognition

Before feeding the time series into into ML models for gross motion classification, the acquired RTLS-based data streams undergo a feature extraction. The raw positional 2D information is expressed in Euclidean distances to each WS, creating layout-insensitive data representations. The input space is completed by the RTLS-acquired unique Operator ID and two time-driven features derived from the sensor timestamp. The operator ID distinguishes workers active within the same production setup, while the local clock measures the interval between consecutive samples from each operator, preserving the timing of task streams performed in parallel. The global clock measures elapsed time from the earliest timestamp in the production setup and represents its overall progression. This data representation is processed by three different ML models with different learning structures.

**LSTM:** A one-layer LSTM architecture is implemented to learn temporal dependencies in workers’ motion data. Input sequences are generated using overlapping sliding windows of 128 samples with a one-sample stride and processed in batches of 64. The network comprises one LSTM layer with a hidden-state size of 256, followed by a fully connected classification layer and softmax activation. The dropout rate is not applied, and the model has 278,536 parameters. Input features are min–max normalised to the range [0,1]. The implementation sets random seeds to 1 and minimises cross-entropy loss using Adam with a learning rate of $10^{-4}$. Training runs for 300 epochs without early stopping, and the model weights are saved after each epoch. The checkpoint with the highest micro-averaged F1-score on the validation set is selected for reporting.
**Industrial DBscan:** The method extends standard unsupervised clustering formulations by incorporating temporal sensitivity and an explicit mechanism for mitigating positional uncertainty [31]. It identifies human-process interactions as dense concentrations of spatio-temporal observations within predefined layout regions and associates the resulting clusters with corresponding industrial resources. The method requires no training phase, although its spatio-temporal hyperparameters must be predefined and assigned statically. Initial clusters are identified using a spatial threshold of ϵ=0.17 m and a minimum number of points of NPts=15. Temporal consecutiveness is enforced using δt=10 Hz. Consecutive interactions are then merged based on a maximum temporal separation of α=1 s and spatial thresholds of β=ϕ=0.5 m. Finally, the resulting clusters are assigned to the corresponding industrial resources.
**Random Forest:** The method uses an ensemble of decision trees to classify workers’ activities from engineered motion features. The optimised configuration comprises 70 trees, applies entropy as the splitting criterion, requires at least three samples to split an internal node, and considers the square root of the available features at each split. Bootstrap sampling and random feature selection promote diversity among the trees and limit overfitting. The final activity class is determined through majority voting across the individual predictions.

The benchmark uses micro-averaged F1 as its overall performance measure. Each test sample contributes equally to this score, so classes with more samples have a greater influence on the result. Precision, recall, and F1 are therefore also reported for each class to show how well the methods recognise less frequent activities. The LSTM and Random Forest use the same training, validation, and test partition, whereas Industrial DBSCAN requires no training.

#### 4.2.2. Neural Networks for Fine Worker Activity Recognition

This second level of WAR recognition benchmarks different neural network learning patterns and data representations. The Transformer architecture uses a feature-extracted representation of IMU data streams. Temporally aligned 3D accelerations and quaternions are segmented using a sliding window of 128 samples with 50% overlap between consecutive windows. For each window and each signal axis, statistical features are extracted in both the time and frequency domains. Time-domain features include mean, variance, and root mean square (RMS), while frequency-domain features include mean frequency and median frequency. This compressed dataset presents 19,278 labelled input samples and 30 features. On the contrary, two deep CNNs leverage the raw dataset of tri-axial accelerations and quaternions with almost one million data samples. Despite their different input representations, all three networks use the same allocation of source recordings to the training, validation, and test subsets and follow the same training protocol. Each input feature is standardised using the mean and standard deviation calculated from its respective training subset, with those statistics then applied to validation and test data. The models use Adam with an initial learning rate of $10^{-3}$, a random seed of 42, and a maximum of 300 epochs. Training stops after 30 epochs without an improvement greater than $10^{-4}$ in validation macro-F1, and the best weights are restored. Networks are benchmarked using the aggregate F1-score, defined as the unweighted mean of the five class-specific test F1-scores, each calculated as the harmonic mean of precision and recall. The following paragraph details the neural network design that shares the Adam algorithm as the parameter optimiser.

**Transformer:** The model adopts an encoder-only Transformer architecture. A one-dimensional convolutional projection with 100 output channels, a kernel size of 3, and a stride of 1 first maps the input sequences. Sinusoidal positional encoding then provides sequence-order information. The encoder applies multi-head self-attention with two heads using learnable query, key, and value projections and scaled dot-product attention. It also includes two residual Add and Norm blocks with batch normalisation and a feed-forward network comprising two linear layers with an inner dimension of 1028 and an intermediate ReLU activation. A final linear layer with softmax activation produces the activity-class probabilities. The Transformer is the smallest benchmarked network, accounting for 76,233 parameters.
**ResNet:** This architecture follows a one-dimensional formulation of the residual learning framework presented in [32]. It begins with a temporal convolutional stem comprising 64 filters with kernel size 7, followed by batch normalisation and ReLU activation. The network body consists of six residual blocks configured with filters (64,64,128,128,256,256), while projection shortcuts are introduced whenever channel width or temporal resolution changes. Classification is performed through global average pooling followed by a dense softmax output layer. With 1,106,181 parameters, this CNN is the biggest.
**SqueezeNet:** We adopt a one-dimensional formulation of the Fire-module design introduced in [33]. An initial temporal convolution followed by max pooling precedes eight Fire modules, each combining a squeeze convolution with kernel size 1 and two parallel expansion branches using kernel sizes 1 and 3. The classification stage applies dropout, a 1D convolution with kernel size 1, global average pooling, and a final softmax classification output layer. This network has 360,325 parameters.

### 4.3. Decision-Support System

**Figure 6:**
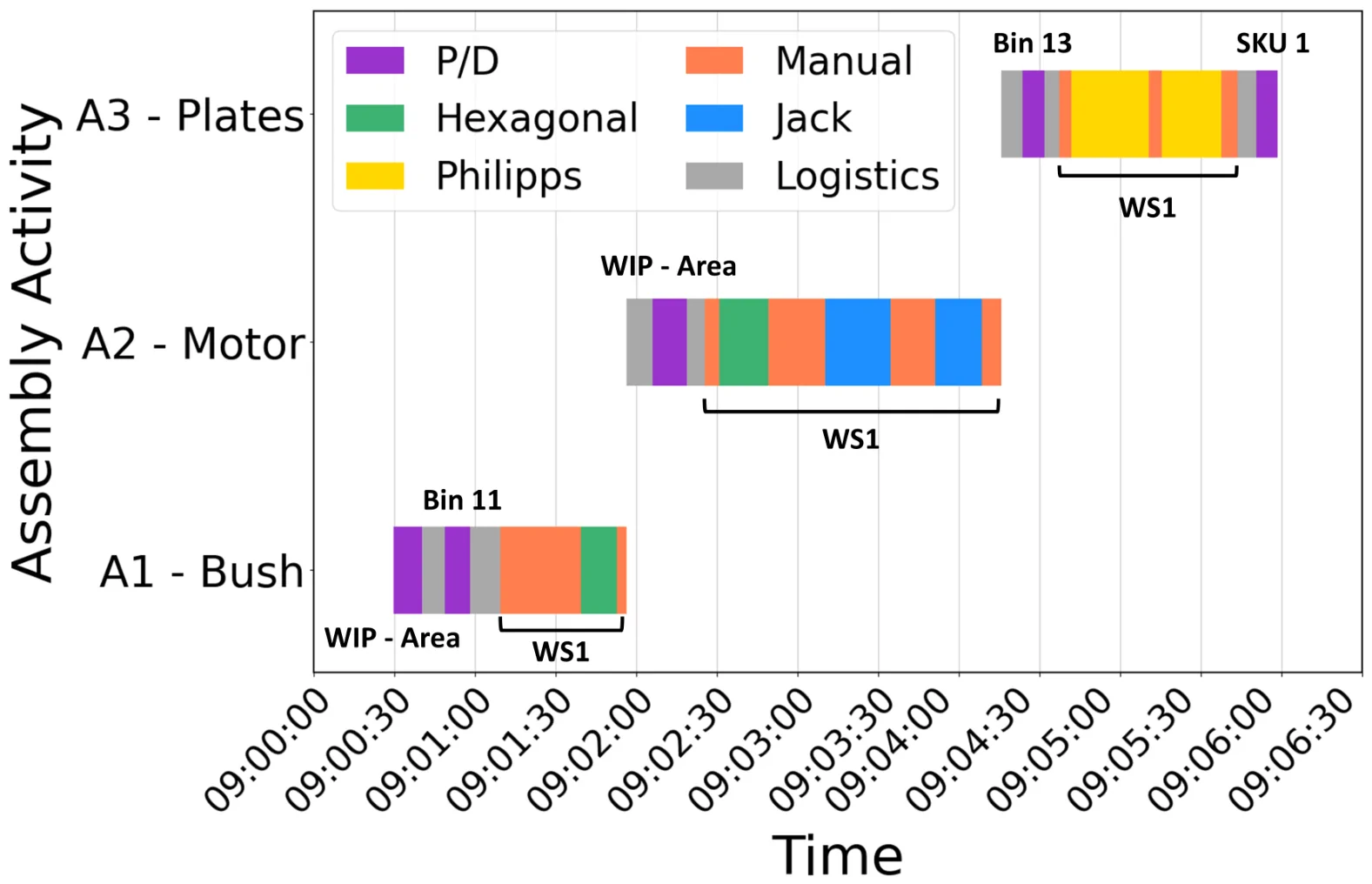
Activity segmentation of human-centric manufacturing systems.

This final CPS layer segments human-centric task executions by exploiting ML-classified RTLS and IMU data streams. For each measurement, the decision-support system queries InfluxDB tables and returns the classified tasks in temporal windows that group consecutive and homogeneous activities. Then, RFID-based process interactions are included to provide essential descriptive information, such as storage locations in P/D events. Figure 6 shows a representative activity segmentation obtained by integrating the data from the three sensing technologies. While RTLS identifies logistics events and the WS where operators execute tasks, IMUs segment the fine-grained assembly tasks within each WS. RFID data streams complete the activity context by detailing P/D locations within supermarket bins and storage entities (e.g., SKU 1).

The identified time-driven events provide enhanced visibility into manufacturing operations and establish a structured basis for monitoring system and operator resilience through different KPIs. In particular, the classified gross motion events support the calculation of the following manufacturing metrics:

- **Industrial Resource Utilisation:** This KPI measures the proportion of time that workstations and machines contribute to manufacturing operations. For example, a resource operating at 80% utilisation while others remain below 30% indicates workload imbalance and potential bottlenecks. These insights support workload reallocation, staffing adjustments, layout improvements, and task-time compliance.
- **From-to charts:** By focusing on human-centric logistic activities, this KPI expresses the frequency, distance, and duration of material handling events between resources. These insights are essential for revealing repetitive or unnecessary trips, which often indicate insufficient buffer capacity, ineffective replenishment strategies, or poor layout design. Beyond productivity, from-to charts support operator resilience by highlighting potential uneven logistic activities. When combined with heavy or difficult-to-handle loads, this imbalance may increase physical strain and ergonomic risk.

Fine-grained WAR events complement manufacturing monitoring by providing the following metrics:

- **Activity duration:** This measures the time required to complete individual tasks at each workstation. Historical records enable supervisors to identify abnormal durations that may indicate emerging bottlenecks. For example, if a task normally requires 30 s but exceeds a 60 s threshold, operators can be reallocated or task sequences revised. This metric also distinguishes value-added from non-value-added time, including waiting, material searching, and part repositioning.
- **Throughput time:** This represents the total elapsed time required for a product to complete all activities. It captures processing, waiting, and material-movement times, providing an indicator of production-flow performance. Increasing throughput times may indicate insufficient staffing, poor workstation synchronisation, or growing queues, while reductions suggest smoother operations.
- **Tool usage:** This evaluates equipment availability and supports Overall Equipment Effectiveness (OEE) assessment. Availability measures the proportion of planned production time during which equipment operates, while OEE performance compares the actual processing rate with the ideal rate. These indicators can inform heuristic scheduling algorithms and minimise delays caused by concurrent equipment demands.
- **Ergonomic evaluation:** Fine-grained WAR segmentation provides the contextual information required to monitor workers’ well-being with a limited set of additional sensors. For instance, physical load analysis may be supported by using weighting sensors to estimate the effort in logistic activities. Additional IMUs may capture body kinematics and classify postural risks against ergonomic assessment worksheet system (EAWS) thresholds, including repeated bending beyond 60°, prolonged twisting, and excessive reaching [34]. Finally, surface electromyography sensors complement this analysis by measuring muscular activation relative to maximum voluntary contraction and identifying fatigue, peak loads, and limb asymmetries.

The following Section 5 evaluates the baseline performance of ML algorithms at both levels of motion granularity.

## 5. Results: Benchmarking and Baseline Models

This Section presents the classification performance of different ML algorithms. While the gross motion benchmarks an LSTM-based neural network with clustering and tree-based models (see Section 5.1), an attention-based network is compared with two CNNs at the fine level (see Section 5.2).

### 5.1. RMS Gross Worker Activity Recognition Performance

The ML models are trained and tested on a remote Jupyter server hosted on a Dell PowerEdge R740 equipped with an Intel Xeon Platinum 8468H processor and 256 GB of RAM. The LSTM and RF use the same dataset, partitioned into training (70%), validation (15%), and test (15%) subsets. All recordings from a given combination of recording day and setup are assigned to one subset, with two exceptions: Day 2, Setup 5 is shared between training and validation, and Day 2, Setup 8 is shared between validation and test. In both cases, the split is made between operators. All recordings from an individual operator within that combination remain in a single subset, preventing the same operator’s time series from crossing subset boundaries and limiting leakage of operator-specific temporal information. The feature extraction discussed in Section 4.2 is performed after the dataset is partitioned. The Gross WAR data repository includes a spreadsheet (e.g., GrossSplit.xlsx) documenting the recording day, setup, operator, and subset assignments needed to reconstruct the exact split. While the recurrent network requires approximately 90 s per training epoch and 38 s for inference, the tree-based algorithm is massively faster, accounting for 140 s and 15 s for training and inference, respectively. In contrast, Industrial DBscan does not require training and takes approximately 72 s per operator in each production setup of the gross motion dataset.

**Figure 7:**
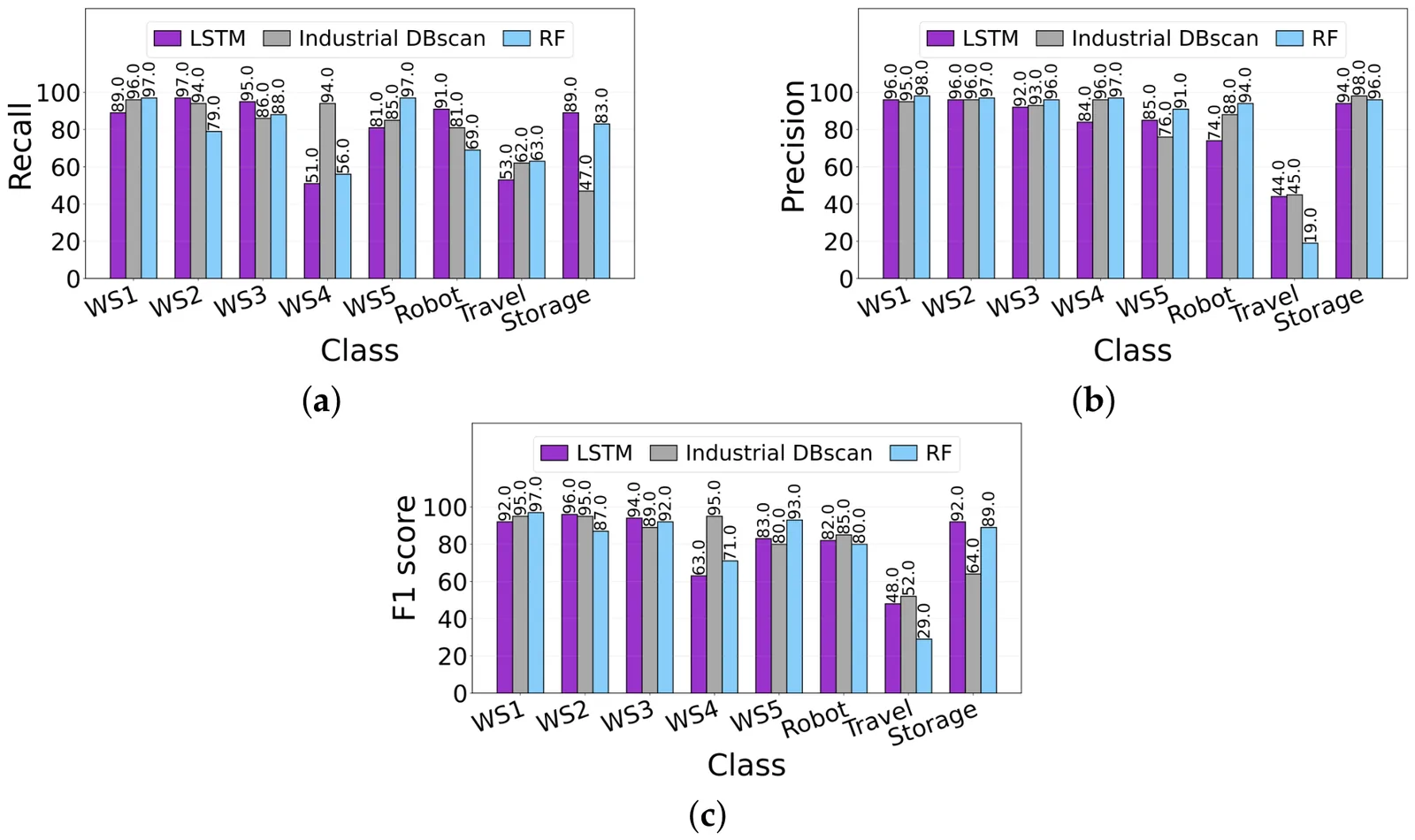
Neural network benchmark for gross WAR: (**a**) class recall; (**b**) class precision; (**c**) class F1-score.

At the aggregate level, Industrial DBscan achieves the highest F1-score of 87%, followed by the LSTM at 86% and the RF at 82%. Figure 7 provides the corresponding class-level recall, precision, and F1-scores. The three approaches perform consistently well for WS1–WS3, with F1-scores ranging from 89% to 97%. Their performance differs more substantially for WS4, WS5, Robot, Travel, and Storage. Industrial DBscan achieves the highest F1-score for WS4 at 95%, compared with 71% for the RF and 63% for the LSTM. The RF performs best for WS5 with an F1-score of 93%, while the clustering algorithm provides the highest result for the Robot class at 85%. The Travel class remains the most challenging for all three approaches. Industrial DBscan achieves an F1-score of 52%, followed by the LSTM at 48% and the RF at 29%. This classification challenge is associated with the proximity of the workstation locations across the production setups, as reported in Table A1. The resulting short transitions reduce the spatial separation between Travel and workstation activities, thereby challenging the resolution and accuracy of the RTLS-based acquisition layer. The class-level results also indicate different error patterns, as the RF achieves a Travel precision of only 19%, while the LSTM and Industrial DBscan exhibit limited recalls of 53% and 62%, respectively. Although Industrial DBSCAN achieves the highest aggregate F1-score (87% versus 86% for the LSTM), the LSTM is selected as the baseline for the intended material-handling application, where recognition of Storage events is prioritised. The LSTM achieves a Storage F1-score of 92%, compared with 64% for Industrial DBSCAN, mainly owing to higher recall (89% versus 47%). This choice entails a trade-off, as Industrial DBSCAN performs better on WS4 (95% versus 63%). A trained LSTM checkpoint may provide a starting point for inference or fine-tuning in a subsequent campaign with compatible input features and activity classes, while Industrial DBSCAN’s spatial and temporal thresholds may require recalibration under different layouts or positioning conditions. This practical consideration does not establish superior LSTM performance on unseen layouts or workers, which requires dedicated evaluation.

### 5.2. Assembly Fine Worker Activity Recognition Performance

For this WAR level, the computational experiments are performed on a high-performance workstation equipped with an AMD Ryzen 9 7950X 16-core processor, 192 GB of RAM, and two NVIDIA RTX 6000 Ada Generation GPUs, each providing 48 GB of dedicated memory. The benchmark compares different learning architectures and input-data representations, as the Transformer operates on feature-extracted motion patterns, whereas the CNN operates on raw IMU data streams (see Section 4.2). The dataset is divided into training, validation, and test subsets using a target ratio of 70%/15%/15%. Full assembly recordings from Days 1–3 are assigned to training, Day 4 to validation, and Day 5 to test, keeping each recording day within one subset. For Participant 3’s basic operations, the continuous recording is segmented into non-overlapping windows based on the mean repetition duration. Complete repetition windows are then randomly assigned to the three subsets while preserving the target split ratio and overall class distribution. These assignments are made before feature extraction and retained across all three models, so each subset contains the same underlying recordings. This controls data allocation, but differences between the Transformer and the CNNs reflect both architecture and input representation and are therefore interpreted as comparisons of complete processing pipelines. Because ResNet and SqueezeNet both use raw IMU signals, their results permit a more direct architectural comparison. The Fine WAR repository includes range.xlsx and DataSplit.txt to support reconstruction of the exact split.

**Figure 8:**
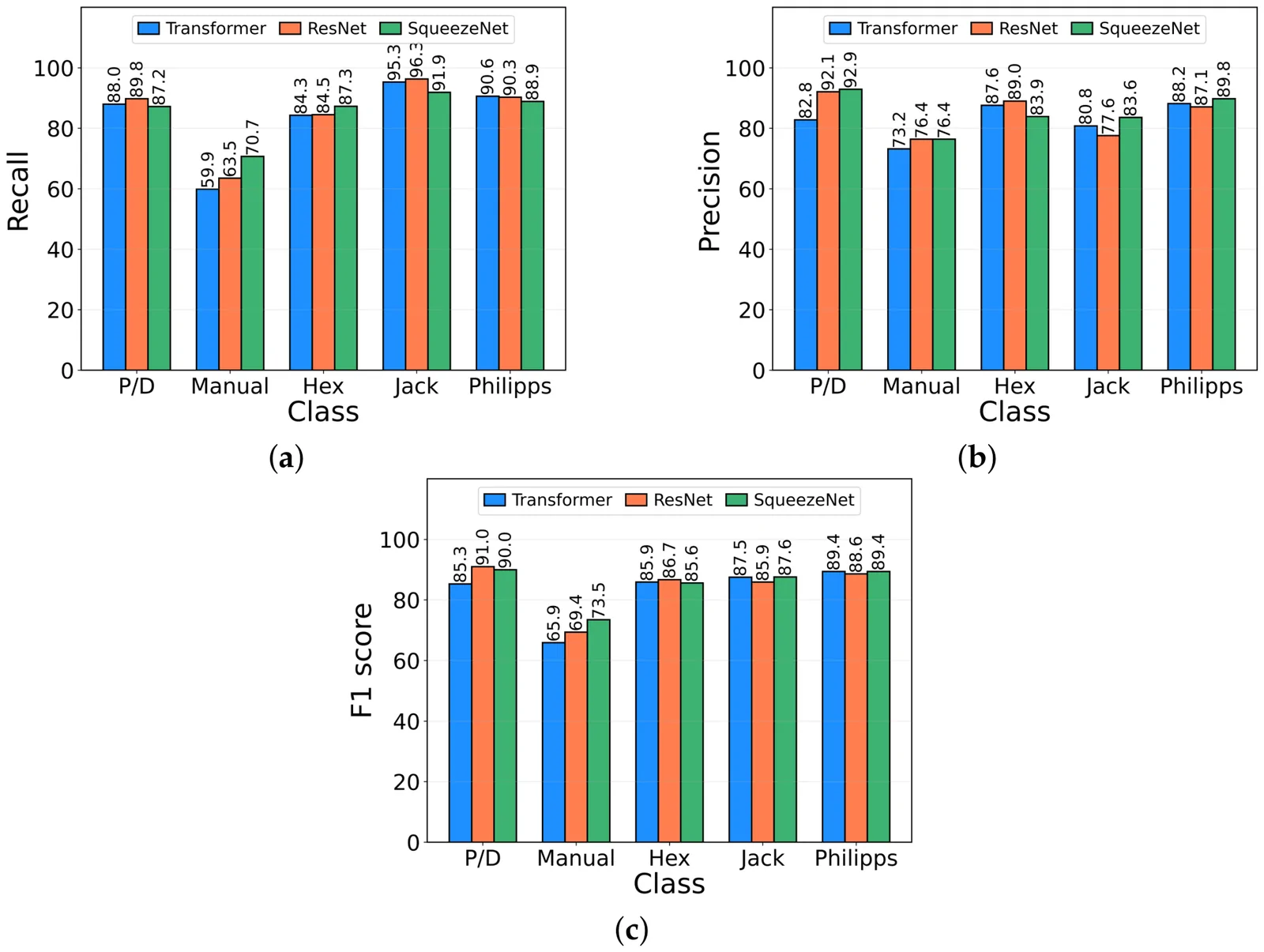
Machine learning models benchmark for fine WAR: (**a**) class recall; (**b**) class precision; (**c**) class F1-score.

The recorded training times are approximately 35 s for the Transformer, 72 s for SqueezeNet, and 117 s for ResNet. On the test set, SqueezeNet achieves the highest aggregate F1-score of 85.2%, followed by ResNet at 84.3% and the Transformer at 82.0%. This result is obtained despite the substantial difference in model size, as ResNet contains approximately three times more parameters than SqueezeNet. Therefore, increasing the network size does not provide a corresponding improvement in classification performance for the considered task. Figure 8 reports the class-level precision, recall, and F1-scores. For the tool-related activities, all three models provide comparatively consistent results, with F1-scores ranging from 85.3% to 91.0%. ResNet achieves the highest F1-scores for P/D and Hex at 91.0% and 86.7%, respectively. SqueezeNet performs best for Jack with 87.6% and matches the highest result for Phillips at 89.4%. These results indicate that the repetitive and recognisable motion profiles associated with tool-related activities can be identified consistently across the evaluated learning architectures. In contrast, Manual is the most challenging class for all three models. Unlike the tool-related classes, Manual does not represent a single motion primitive but combines heterogeneous operations, including component alignment, fastener preparation, hand-based adjustments, tool handling, and transitions between consecutive assembly tasks. These actions are frequently short, weakly repeatable, and partially overlap with neighbouring activity windows. Their execution also varies across operators in terms of speed, posture, and movement trajectory, which reduces their separability from adjacent activities. Nevertheless, SqueezeNet achieves the highest Manual recall of 70.7% and an F1-score of 73.5%, compared with 69.4% for ResNet and 65.9% for the Transformer.

Based on its aggregate performance, compact architecture, shorter training time than ResNet, and stronger recognition of the most challenging class, SqueezeNet is selected as the baseline model for fine-grained WAR.

## 6. Conclusions

This study addresses the limited reproducibility and production readiness of WAR in human-centric manufacturing. Two multimodal datasets capture complementary motion levels under realistic operating conditions. The RMS Gross WAR dataset contains 814,065 RTLS-based positioning samples from eight reconfigurable manufacturing setups, each performed twice, and involves more than 40 workers. The Assembly Fine WAR dataset combines two IMUs with a RFID-based smart glove during 50 complete pump-assembly cycles performed by three workers. It provides approximately 7 h of recordings and more than one million samples. A modular CPS connects these sensing configurations to benchmarked ML models and a decision-support layer that converts classified activities into time-driven operational indicators.

On the one hand, the gross WAR benchmark a LSTM neural network with the Industrial DBscan and a RF classifier. Despite the fact that the clustering algorithm achieves the highest F1-score of 87%, the recurrent network with an F1-score of 86% is selected as the gross WAR baseline because it achieves a higher F1-score for Storage (92% versus 64%), supporting the intended material-handling analysis. Moreover, the LSTM does not require the same use-case-specific adjustment of spatio-temporal clustering parameters, facilitating future use-case applications. The travel remains the most difficult gross activity, with F1-scores between 29% and 52%, reflecting the limited spatial separation between short movements and neighbouring workstation activities. On the other hand, SqueezeNet is the baseline model for this Assembly Fine WAR dataset. Besides outperforming the F1-score of both Transformer and ResNet networks, it achieves the strongest result for the heterogeneous Manual class, with an F1-score of 73.5%. Tool-related activities are recognised more consistently, with class-level F1-scores ranging from 85.3% to 91.0%. These findings indicate that compact convolutional models provide an effective balance between classification performance and model size for the considered assembly task. Beyond dataset availability, the findings provide three broader insights for manufacturing WAR. First, transition-rich activities represent the main observed recognition bottleneck across both motion levels. Second, baseline selection should account for class-level operational relevance and deployment constraints rather than aggregate F1-score alone. Finally, integrating gross and fine-grained motion monitoring establishes a reusable framework for translating heterogeneous sensing data into operational indicators of manufacturing performance and worker resilience.

Further research will focus on extending the datasets with additional subjects and activity classes for both levels of motions and further evaluating generalisation across different industrial setups. Sensitivity analyses will assess each model across multiple splits that preserve recording and operator boundaries, with repeated training under different random seeds for the learned models. Reporting the mean and standard deviation of aggregate and class-specific F1-scores will show how sensitive the results are to data allocation and training randomness and whether the observed differences between models persist. 

## Figures and Tables

**Table 1:** Overview of Worker Activity Recognition levels.

WAR Level	Target	IoT	Sampling	Cost
Gross RMS	Detection of proximity to workstations and logistic events	RTLS	≥10 Hz	5000 £ fixed 120 £/anchor 80 £/tag
Fine Assembly	Segmentation of assembly tasks	IMU RFID	≥52 Hz	120 £/IMU 200 £/RFID

## Data Availability

The datasets generated and analysed during this study are publicly available in the Zenodo repository at https://doi.org/10.5281/zenodo.23266446 (record: https://zenodo.org/records/23266446 (accessed on 18 October 2026)).

## Appendix A

This appendix specifies the absolute indoor positions of industrial resources and the number of workers across production setups for the RMS Gross WAR dataset.

**Table A1:** Absolute positions of industrial resources in different production setups.

		WS1		WS2		WS3		WS4		WS5		Robot	
**Setup**	**Workers**	X	Y	X	Y	X	Y	X	Y	X	Y	X	Y
1	6	4.57	3.35	4.43	1.22	6.76	4.13	6.56	2.34	6.23	1.03	6.37	3.12
2	6	4.40	2.79	6.23	4.29	6.17	1.39	4.70	5.12	9.82	3.26	8.44	3.36
3	5	8.20	3.27	6.35	3.96	6.63	1.84	4.94	1.06	4.29	1.73	4.05	3.08
4	4	5.00	3.11	6.24	4.23	6.10	2.03	4.51	5.21	8.87	3.61	9.01	1.86
5	5	4.50	3.31	4.32	1.29	6.23	4.51	5.85	2.08	8.25	3.77	8.04	1.75
6	4	4.57	3.35	4.43	1.22	6.76	4.13	6.56	2.34	6.23	1.03	6.37	3.12
7	5	4.75	0.91	4.30	3.61	7.34	4.25	5.97	3.82	4.78	5.07	6.30	5.70
8	6	4.53	3.37	6.13	2.46	6.34	4.21	4.56	0.88	8.72	3.19	7.98	3.42
